# Challenges, facilitators and barriers to screening study participants in early disease stages-experience from the MACUSTAR study

**DOI:** 10.1186/s12874-021-01243-8

**Published:** 2021-03-17

**Authors:** Jan Henrik Terheyden, Charlotte Behning, Anna Lüning, Ludmila Wintergerst, Pier G. Basile, Diana Tavares, Beatriz A. Melício, Sergio Leal, George Weissgerber, Ulrich F. O. Luhmann, David P. Crabb, Adnan Tufail, Carel Hoyng, Moritz Berger, Matthias Schmid, Rufino Silva, Cecília V. Martinho, José Cunha-Vaz, Frank G. Holz, Robert P. Finger, on behalf of the MACUSTAR consortium, H. Agostini, F. Bandello, P. Basile, C. Behning, M. Berger, A. Binns, M. Böttger, C. Bouchet, J. E. Brazier, T. Butt, C. Carapezzi, J. Carlton, A. Carneiro, A. Charil, R. Coimbra, D. P. Crabb, J. Cunha-Vaz, C. Dahlke, H. Dunbar, M. Durbin, R. P. Finger, E. Fletcher, H. Floyd, R. Hogg, F. G. Holz, C. Hoyng, J. Krätzschmar, L. Kühlewein, M. Larsen, S. Leal, U. Luhmann, A. Lüning, C. Martinho, B. Melício, S. Mohand-Said, S. Nunes, M. Parravano, D. Pauleikhoff, M. Pfau, S. Pondorfer, S. Poor, S. Priglinger, D. Rowen, G. S. Rubin, J. Sahel, D. Sanches Fernandes, C. Sánchez, M. Saßmannshausen, M. Schmid, S. Schmitz-Valckenberg, H. Schrinner-Fenske, R. Silva, A. Skelly, E. Souied, G. Staurenghi, L. Stöhr, D. Tavares, D. J. Taylor, J. H. Terheyden, S. Thiele, A. Tufail, J. Werner, G. Weissgerber, L. Wintergerst, C. Wojek, N. Zakaria

**Affiliations:** 1 Department of Ophthalmology, University Hospital Bonn, Bonn, Germany; 2 Institute for Medical Biometry, Informatics and Epidemiology, University Hospital Bonn, Bonn, Germany; 3 Association for Innovation and Biomedical Research on Light and Image, Coimbra, Portugal; 4 Bayer AG, Berlin, Germany; 5 Novartis Pharma AG, Basel, Switzerland; 6 Roche Pharmaceutical Research and Early Development, Translational Medicine Ophthalmology, Roche Pharma Research and Early Development, Roche Innovation Center, Basel, Switzerland; 7 Division of Optometry and Visual Sciences, School of Health Sciences, City, University of London, London, UK; 8 Moorfields Eye Hospital, London, UK; 9 Radboud University Medical Center, Nijmegen, Netherlands; 10 University of Coimbra, Coimbra Institute for Clinical and Biomedical Research (iCBR), Faculty of Medicine, Coimbra, Portugal; 11 Ophthalmology Department, Centro Hospitalar e Universitário de Coimbra (CHUC), Coimbra, Portugal

**Keywords:** Early disease stages, Age-related macular degeneration, Cohort study, Screening, Recruitment

## Abstract

**Background:**

Recruiting asymptomatic participants with early disease stages into studies is challenging and only little is known about facilitators and barriers to screening and recruitment of study participants. Thus we assessed factors associated with screening rates in the MACUSTAR study, a multi-centre, low-interventional cohort study of early stages of age-related macular degeneration (AMD).

**Methods:**

Screening rates per clinical site and per week were compiled and applicable recruitment factors were assigned to respective time periods. A generalized linear mixed-effects model including the most relevant recruitment factors identified via in-depth interviews with study personnel was fitted to the screening data. Only participants with intermediate AMD were considered.

**Results:**

A total of 766 individual screenings within 87 weeks were available for analysis. The mean screening rate was 0.6 ± 0.9 screenings per week among all sites. The participation at investigator teleconferences (relative risk increase 1.466, 95% CI [1.018–2.112]), public holidays (relative risk decrease 0.466, 95% CI [0.367–0.591]) and reaching 80% of the site’s recruitment target (relative risk decrease 0.699, 95% CI [0.367–0.591]) were associated with the number of screenings at an individual site level.

**Conclusions:**

Careful planning of screening activities is necessary when recruiting early disease stages in multi-centre observational or low-interventional studies. Conducting teleconferences with local investigators can increase screening rates. When planning recruitment, seasonal and saturation effects at clinical site level need to be taken into account.

**Trial registration:**

ClinicalTrials.govNCT03349801. Registered on 22 November 2017.

## Background

Recruiting asymptomatic participants with early disease stages into clinical or epidemiological studies is challenging because these individuals might not be aware of their disease and their perceived disease burden is often low. In order to overcome these challenges, careful planning of screening and recruitment activities is crucial. This includes careful evaluation of screening and recruitment facilitators as well as barriers. A number of studies have reported factors that impact recruitment at different levels [1–7], but knowledge about how to best identify the specific target population of asymptomatic participants with early disease stages into studies remains limited. Against this background we assessed the recruitment process and any measures which impacted screening numbers in a study of early, largely asymptomatic stages of age-related macular degeneration (AMD). Our goal was to retrospectively identify facilitators and barriers to screenings from a sponsor’s perspective in a multi-center cohort study of early disease stages.

The reason for addressing early AMD stages in clinical research today is to reduce the signicficant burden of late-stage AMD by developing novel interventions that stop or delay progression from early AMD stages to late AMD and prevent potentially irreversible loss of vision which make late AMD a leading cause of visual loss in industrialised countries [8, 9]. Early stages of AMD progress slowly at an estimated rate of 5–20 per 100 person-years to late AMD [10] and frequently cause no or only little symptoms [11, 12]. Similar to other early disease stages such as early Alzheimer’s disease, prediabetes or pre-clinical cancer [13–15], individuals with early stages of AMD are frequently not aware of their disease [16]. This makes it important to investigate which measures facilitate or impede screening activities for clinical studies of early AMD as identified factors are of potential relevance to other studies recruiting asymptomatic participants. We herein report the impact of both facilitators and barriers to screening participants for the MACUSTAR study, a multi-national cohort study focusing on the most high-risk early stage of AMD (“intermediate AMD”) from a sponsor’s perspective [17].

## Methods

### The MACUSTAR study

The MACUSTAR study is a multi-centre cohort study focusing mainly on “intermediate AMD”, a high-risk type within the early AMD stages. The main study objective is the development of new candidate endpoints for intermediate AMD clinical trials. For this purpose, participants at all AMD disease stages (no, early, intermediate and late AMD) undergo a battery of functional tests and imaging procedures and several patient-reported outcome measures are administered. The majority of participants of the MACUSTAR study has intermediate AMD and was recruited at 20 study sites while the other groups (early AMD, late AMD, no AMD) were recruited only at five study sites. More details on the study protocol including the eligibility criteria, visit schedule, outcome measures and their assessment, confounders, sources of bias and sample size considerations have been published previously [18].

Recruitment for the MACUSTAR study started in March 2018 and lasted for 87 weeks. Patients were screened and recruited at 20 ophthalmological clinical sites in seven European countries (Denmark, France, Germany, Italy, Netherlands, Portugal and United Kingdom). Five of them were academic core partners within the MACUSTAR consortium, the other sites were affiliated with the consortium and members of the European Vision Clinical Research Network (EVICR.net). To facilitate planning of screenings, all sites confirmed their ability to recruit a minimum of 20 individuals into the MACUSTAR study before study initiation; the core partners agreed to a higher target of 40–70 recruited participants. Herein we retrospectively analyse and report the impact of screening measures, and other factors found to be either facilitators or barriers to screening participants.

All institutional ethic committees approved the study and participants gave written informed consent prior to participation. The MACUSTAR project receives funding from the European Union Innovative Medicines Initiative (IMI2) Horizon 2020 programme. It has been registered at the website clinicaltrials.gov with the identifier NCT03349801. Inclusion criteria for this analysis were individuals screened for the MACUSTAR study with the screening diagnosis intermediate AMD, a high-risk type of the early AMD stages (determined at the clinical site). Study inclusion at all study sites was based on the evaluation and confirmation of AMD diagnosis by a central reading centre, as described previously [17]. Exclusion criteria were missing informed consent, participation in any of the other MACUSTAR study groups (early, or late AMD or control group) or relocation to another clinical site within the time of the study. The MACUSTAR clinical study is managed by the academic clinical research organization AIBILI (Association for Innovation and Biomedical Research on Light and Image, www.aibili.pt) and monitored by the European distributed infrastructure network ECRIN-ERIC (www.ecrin.org).

### Qualitative evaluation of screening measures

Screening strategies and measures were planned centrally by a coordination team and then implemented through AIBILI and ECRIN-ERIC across all sites. In order to be able to systematically assess the impact of any of these on screening numbers, we extracted all relevant information retrospectively from the study protocol, protocol amendments, clinical site communications such as newsletters and briefings, status reports, meeting minutes and emails from March 2018 to March 2020. All factors that may have contributed to screening rates were compiled and assigned to time periods and clinical sites within the recruitment phase of the study where they had been implemented. Time is measured in weeks since the first site opened. Furthermore, we conducted four in-depth interviews with personnel actively involved in the study (clinical project managers, study site coordinators and research personnel) to identify the most relevant screening factors based on the available screening numbers and factors previously identified. The interviews consisted of two parts to identify additionally relevant factors: Firstly, all interviewed persons were asked to name which factors (a) facilitated screenings or (b) impeded screenings. Secondly, the factors were ranked by the perceived impact on screening numbers by each person interviewed. All persons were interviewed once and only qualitative methods were applied during this step.

### Screening data compilation

Due to the availability of devices, ethics approvals, contracting and the necessity to implement the upcoming European Union General Data Protection Regulation in 2018, the first participants were screened at different time points at the participating study sites. After completion of recruitment, data from the electronic case report form and imaging data were collected and cleaned as reported in the study protocol [18]. Screening numbers and recruitment numbers were compiled per clinical site and per week. We assigned week 1 to the first week of recruitment at the first site and used a global consecutive numbering of weeks for all sites. Factors that were considered relevant in the qualitative evaluation (listed chronologically and preceded by a # sign in the results) were assigned to specific clinical sites and to specific time periods within the screening number database based on where and when they were implemented or occurred.

### Statistical modelling

We explored the relationship between different screening factors and screening numbers per week at each clinical site. The inter-correlation of recruitment factors was assessed using Pearson correlation coefficients. When two variables correlated with r > 0.8, only the factor more strongly associated with screening numbers in qualitative evaluation was used for further analyses. To account for repeated measurements within the study sites, a generalized mixed-effects model was built to investigate the effect of screening factors on the screening numbers per site. As the official start of the recruitment phase was scheduled at different time points for each clinical site, a random intercept depending on the site’s activity status was included. Each selected screening factor entered the model as a fixed effect. To capture a possible time-trend, the week number (measured in weeks since first site has opened) was also considered as a fixed linear effect. Since the screening numbers can be treated as count data, we used the Poisson family with a logarithmic link function. Associations between screening numbers and factors contributing to screening numbers are presented in terms of relative risk increases (exp(β)) with 95% confidence intervals, where β denotes the coefficient estimate obtained from the mixed-effects Poisson model.

The analyses were performed with the software R, version 3.6.1 (R Core Team 2020, Vienna, Austria), using the packages lme4 and MuMIn [19, 20].

## Results

**Fig. 1:**
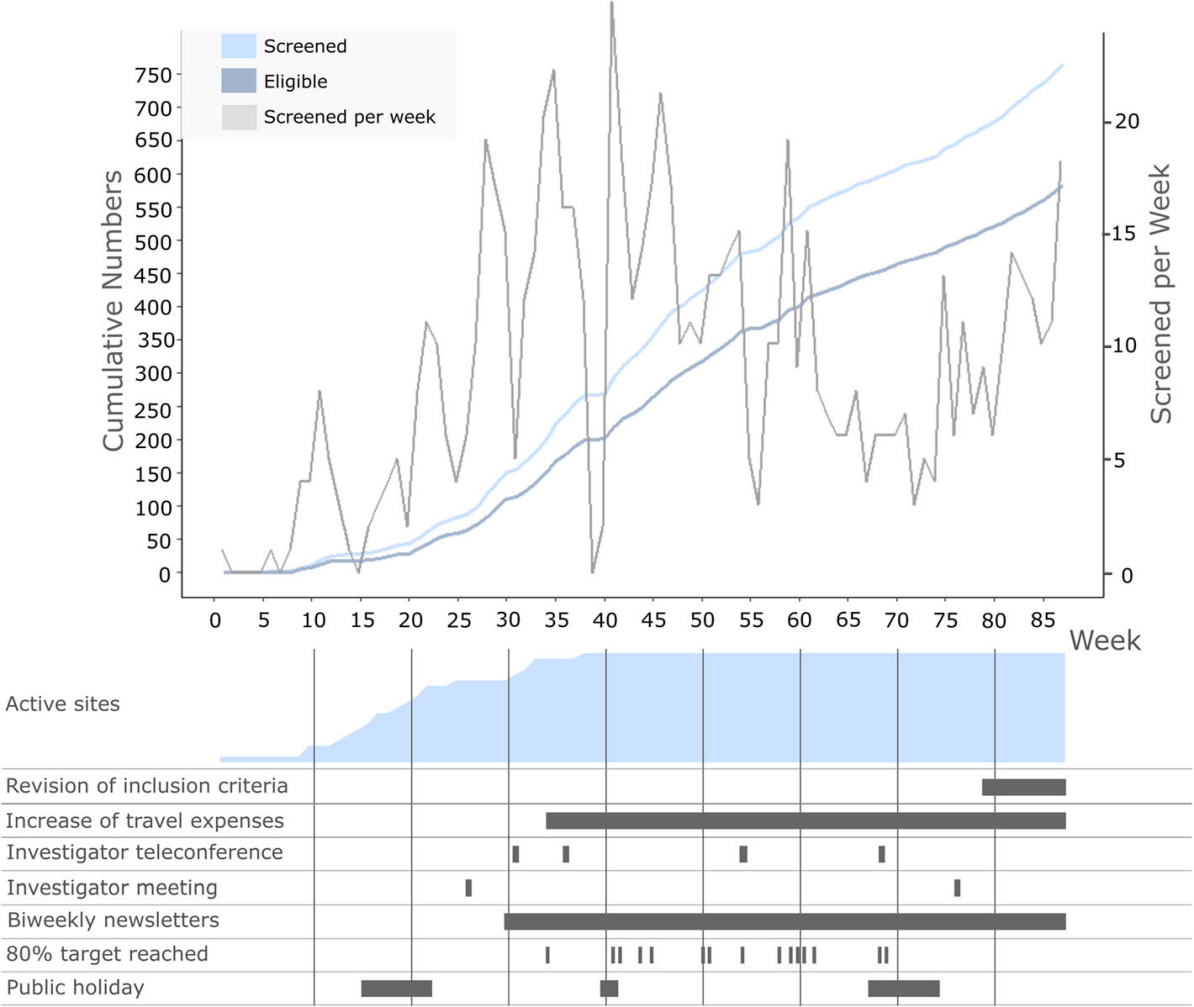
Participants screened and participants eligible for the MACUSTAR study with intermediate age-related macular degeneration as well as number of active sites and factors impacting screenings, displayed per week since start of recruitment at the first study site (80% target reached refers to individual clinical sites; all other factors are global). The blue curves represent cumulative numbers; the grey curve represents numbers per week

The overall number of screenings for the intermediate AMD group was 767 in 87 weeks. One participant was excluded from the analysis due to relocating after the screening visit. The last site was opened for recruitment 37 weeks after the first site (Fig. 1). The mean screening rate was 0.6 ± 0.9 screenings per week among all sites. At total of 584 participants of the 766 individuals with intermediate AMD included in the analysis (76%) were included in the MACUSTAR study.

### Qualitative evaluation

Twenty factors with a possible impact on patient screenings were identified at global study level (Table 1). While some of them occurred continuously, others were linked to specific periods of time.

**Table 1:** Relevant global screening factors identified for the MACUSTAR study in qualitative evaluation ordered by estimated magnitude of impact on screening numbers

Screening measures	Factors prioritized in qualitative interviews*	Other factors
***All sites***	Change of inclusion criteria (opening for individuals with unilateral intermediate disease) (#8)	Dissemination material (patient flyer, referral letter, study procedure flyers, sample visit schedules)
	Increase of participant travel expenses reimbursement (#3)	Distribution of study newsletter
	Investigator teleconferences (#6)	
	Investigator meetings (conferences) (#7)	Letter of appreciation for clinical sites at recruitment start
	Increase of study newsletter frequency (monthly to biweekly) (#4)	Implementation of a clinical site questionnaire to identify unsolved issues
	Regular coordination teleconferences with the project management and monitors (#5)	
***Single sites***		Pre-screening lists
		Individual contacts with investigators (e-mail, phone, in person)
		Individual contacts with site coordinators
		Appearing in the newsletter as a “top recruiter”
**Interacting factors**	Public holiday (#2)	Competitive recruitment
	Reaching a high proportion of the initial “recruitment target” or exceeding this target (#9)	Communication of recruiting problems by individual sites
	Successive initiation of screening activity (#1)	Problems with study devices at individual sites
	Consortium core membership (#10)	

* only global factors that could be assigned to specific time periods were allowed

Factors preceded by a # sign were considered relevant in the qualitative evaluation and are displayed in the ranking order obtained in the qualitative evaluation

Screenings in the MACUSTAR study proceeded in three phases. During an *initiation period* (weeks 1–25), the overall screening trend increased and most participating clinical sites were successively opened for recruitment (factor #1). The weekly screening numbers increased noticeably after the summer holiday season of 2018 (#2). Several screening / recruitment measures implemented continuously throughout the MACUSTAR study were initiated in this period, including the recruitment of patients from pre-screening lists, use of dissemination material and a study newsletter as well as individual contacts with investigators (i.e. phone calls and emails to the principal investigator).

In the ensuing *execution period* (weeks 26–60) weekly screening numbers varied more (range: 0–25 total screenings per week). The implemented measures at the beginning of the *execution period* included an increase of participant travel expenses reimbursement from initial EUR 50 per visit to EUR 75 per visit (#3), an increased MACUSTAR newsletter distribution frequency from monthly to biweekly (#4) and the initiation of regular coordination teleconferences with the project management and monitors (#5). Three teleconferences with the principal investigators (#6) were conducted during the *execution period* and screening numbers increased after these teleconferences. The teleconferences were used to provide data from recent interim analyses to the study staff (principle investigators, study coordinators, study technicians) as well as to allow anyone to ask questions and share approaches on common organizational hurdles, such as the organization of the study schedule, feedback on why screenings failed or pitfalls in the recruitment. Two in person investigator meetings (#7) were also followed by an increase in weekly screenings. The recruitment period was extended beyond the initial end in week 48 until week 87 in order to meet recruitment targets. The two lowest weekly screening rates in the *execution period* (weeks 39 and 56) coincided with Christmas 2018 and the planned end of recruitment before being extended.

The third phase of screenings was a *transition period* (weeks 61–87). It was characterized by more steady screening rates. The number of weekly screenings decreased in the summer holiday season 2019 but increased noticeably afterwards. A change in the inclusion criteria (#8), which opened up recruitment for individuals with unilateral intermediate AMD, was associated with an increase of the screenings at the end of the recruitment period before the transition to the follow-up phase of the study.

At the single clinical site level, the cumulative screenings followed two different patterns. At eight sites, this development increased continuously while at 12 sites, a saturation of the screening rates towards the end of the recruitment period was observed (#9). The core partner sites reached higher recruitment rates (overall median recruitment per site: 66 people) than the other clinical sites (overall median recruitment per site: 29 people; #10).

### Variable selection process

Three of the 10 global variables identified (Table 1) were highly correlated (#3 – #5; increase of travel expenses reimbursement, increase of newsletter frequency, initiation of regular coordination teleconferences with the project management and monitors). In the in-depth interviews (see above), higher travel expenses reimbursements were considered to have the largest impact on the overall screening numbers and we therefore included this factor in the multivariable model only. Thus, we identified the following eight parameters for further statistical evaluation in a multivariable model: Modification of inclusion criteria, increase of participant travel expenses reimbursement, organization of investigator teleconferences and meetings in person, public holidays, saturation of screening numbers (80% of overall recruitment per site), week and being a core partner in the MACUSTAR consortium.

### Multivariable screening number model

A mixed-effects model including the variables identified qualitatively, excluding highly correlated variables (factors #1 – #3, #6 – #10, Table 1) was fitted to the screening data. The participation at investigator teleconferences, public holidays and reaching a high proportion (80%) of the site recruitment target showed strong associations with screening rates at an individual site level (Table 2). The conditional R^2^ value of the model was 0.95 [19, 20]. According to this modelling approach, expected screening numbers increased by the factor exp.(β) = 1.466 (95% CI [1.018–2.112]) after investigator teleconferences were implemented, decreased by the factor exp.(β) = 0.446 (95% CI [0.367–0.591]) during public holidays and decreased with a factor of exp.(β) = 0.669 (95% CI [0.367–0.591]) after a site reached 80% of their recruitment target (after adjusting for the other factors included in the model). This is in line with the average screenings per week, which increased from 0.56 to 0.97 at the time of investigator teleconferences. They decreased from 0.65 to 0.29 during holiday periods and from 0.67 to 0.40 when individual sites reached 80% of their recruitment target.

**Table 2:** Model parameters for the screening numbers per week in a multivariable generalized mixed-effects model (Poisson family with logarithmic link function)

Predictor	β coefficient*	exp (β)*	95% interval for exp(β)*	***p*** value
Revision of inclusion criteria	0.186	1.204	(0.881–1.647)	0.243
Increase of travel expenses reimbursement	0.149	1.161	(0.859–1.570)	0.331
Investigator teleconferences	0.382	1.466	(1.018–2.112)	0.0398
Investigator meetings	−0.084	0.919	(0.705–1.199)	0.534
Core partner site	0.379	1.460	(0.254–8.392)	0.671
Reaching 80% of site recruitment target	−0.357	0.699	(0.542–0.903)	< 0.001
Public holidays	−0.763	0.466	(0.367–0.591)	< 0.001
Week	−0.006	0.994	(0.986–1.003)	0.202
Intercept	−4.19	0.015	(0.005–0.045)	

* adjusted values. No evidence for overdispersion was found (dispersion parameter: 1.0098, *p* = 0.3820 [19]). Only a shared fixed intercept is added when the site is inactive (β_0_ = − 4.19, exp.(β_0_) = 0.015, 95% CI [0.005–0.045]). Random intercepts α for active clinical sites ranged from 3.42 to 4.35

## Discussion

In the MACUSTAR study, we successfully recruited a large cohort of participants with early, mostly asymptomatic AMD stages and found that constant interaction with clinical sites including newsletters, investigator meetings, teleconferences, individual contacts and troubleshooting improve overall recruitment performance. Out of this flurry of activities, however, regular investigator teleconferences were the only measure which was significantly associated with increased screenings at site level. As was to be expected, public holidays were associated with decreased screening performance. Sites slowed down screenings when they reached 80% of their recruitment target. In summary, regular interactions with the site investigators are crucial for a smooth recruitment, and this should likely be increased once sites need to recruit the last 20% as this was when screenings slowed down again.

Failure to recruit a sufficient number of participants in any study can have dire consequences. In an evaluation of two funding agencies, 36% of 195 trials reached less than 80% of their recruitment targets, resulting in a reduced power which had medical, scientific, financial and ethical implications [21, 22]. In addition, low recruitment is a frequent cause for early termination of clinical studies [23]. Careful recruitment planning is therefore an absolute necessity in all clinical studies. In MACUSTAR, no single measure resulted in successful completion of recruitment alone. This is in keeping with available literature where it has previously been noted that only a combination of recruitment measures can lead to successful completion of study recruitment [24].

A review and meta-analysis of recruitment facilitators identified telephone reminders to non-responding candidate participants as a significant facilitator of recruitment to randomized controlled trials [1]. We assume that one of the mediators of this effect was that participants were encouraged to allocate their resources in ways that supported the studies. Similarly, teleconferences with the investigators had a significant positive impact on the MACUSTAR screenings in a multi-site setting. In our experience, teleconferences as well as individual calls allow for a personal relationship and multi- or bidirectional conversations with the site staff on e.g. goals and site-specific difficulties. It also supports peer group learning and creates a common sense of responsibility for the study. In contrast to our findings, Caldwell et al. did not find significantly increased recruitment when keeping increased contact with investigators [2].

Screening rates for the MACUSTAR study dropped significantly during public holidays. This result has not been reported in the available literature [1–3, 5, 21, 25–29] but seems self-evident as facilities are closed during holidays. Gkioni and colleagues reviewed models for the prediction of recruitment when trials are designed [6]. They described that seasonal variations were considered by only 17% of the predictive models found in the literature. We observed high absolute increases in weekly screenings shortly after the end of holiday periods. This finding could be of strategic value for the initiation of recruitment measures in other clinical studies. We assume that facilitating recruitment with new measures could be particularly effective after public holidays.

Besides the assumed influence of teleconferences and public holidays, we observed significant saturation effects of screening numbers in the MACUSTAR study. These would be expected in a study with committed recruitment goals for each clinical site. However, with competitive recruitment in the MACUSTAR study this was an unexpected finding and future research is needed to further assess this effect. In terms of practical implications, sponsor contact should be increased for clinical sites which have almost reached their recruitment target.

Besides these global factors which were present or implemented across all clinical sites in this study, site specific factors such as existing referral networks or a history of clinical research projects are likely to impact screenings numbers and recruitment as well. Unfortunately, it is impossible to assess the impact of any site-specific factors in a systematic fashion as they cannot be quantified across sites.

The main strengths of our analysis include its qualitative and quantitative research methodology, its focus on multi-centre epidemiological research and its inclusion of recruitment factors also identified by previous studies following a thorough review of the literature. We focused our analysis on screening numbers on a site level. Recruitment was not directly assessed in our model since recruited participants out of the pool of screenings were determined by a central reading centre, not by the local investigator. The main limitation of our study is its retrospective character and thus limited generalizability to other studies. As the very few previous studies on recruitment facilitators were done in controlled interventional trials, our results from this observational study have to be interpreted with caution but add to the existing literature. In addition, our analyses provide valuable information in particular relevant to studies recruiting difficult to recruit populations such as early and asymptomatic disease stages [30].

In conclusion, many different facilitators and barriers likely interacted during the recruitment phase of the MACUSTAR study, a multi-site cohort study of early stages of AMD. Regular teleconferences with site investigators increased while public holidays and screening activity saturation at individual clinical sites decreased screening performance. These factors should be given special attention in the design and conduction of future studies as well as selection of clinical sites in particular when recruiting participants with early and largely asymptomatic disease stages.

## Data Availability

The data proving the main findings of the study are contained within the manuscript. The overall dataset used for analysis is available from the MACUSTAR consortium and the MACUSTAR data access committee upon reasonable request (mail@macustar.eu).

## Abbreviations

AIBILI: Association for Innovation and Biomedical Research on Light and Image
AMD: Age-related macular degeneration
CI: Confidence interval
EVICR.net: European Vision Clinical Research Network
